# Reports of occupational dermatosis in the state of Espírito Santo from 2007 to 2016

**DOI:** 10.47626/1679-4435-2021-587

**Published:** 2021-04-30

**Authors:** Priscila Resende Marques, Renan Barroso Soares

**Affiliations:** 1 Medicina do Trabalho, Escola Superior de Ciências da Santa Casa de Misericórdia de Vitória, Vitória, ES, Brazil; 2 Química, Instituto Federal do Espírito Santo, Vila Velha, ES, Brazil; 3 Engenharia, Faculdade Multivix, Vitória, ES, Brazil

**Keywords:** occupational dermatitis, disease reporting, causality

## Abstract

**Introduction:**

Occupational dermatoses account for a large fraction of work-related illnesses, and have significant economic and social repercussions. Though these conditions are subject to mandatory reporting, they are often underdiagnosed, and have only been scarcely studied in Brazil.

**Objectives:**

To describe the profile of workers affected by occupational dermatosis based on reports sent to the Information System on Notifiable Diseases [Sistema de Informação de Agravos e de Notificação (SINAN)], and provide evidence to support disease prevention initiatives.

**Methods:**

The following data were collected from incident reports sent in the state of Espírito Santo, from 2007 to 2016: city, year, age of worker, education level, gender, causal agent, race, occupation, lesion site, and need for medical leave. Data were analyzed using Microsoft Excel, using frequency and percentage statistics.

**Results:**

A total of 340 incidents were reported in the state of Espírito Santo, which most cases (79%) occurring in the city of Atílio Vivácqua. The highest number of cases involved female workers aged 20 to 29 years, with complete primary and incomplete secondary education, in unskilled occupations such as domestic work, and were associated with exposure to chemical agents such as chlorine, detergents, and latex. The lesion site was reported in fewer than 2% of cases. At least 25% of affected workers required medical leave.

**Conclusions:**

The individuals most frequently affected by occupational dermatosis were women, aged 20 to 29 years, with complete primary education, in unskilled occupations. There is a need for greater investment in the education of unskilled workers, especially those who perform cleaning services.

## INTRODUCTION

The growth of the global economy has produced significant changes in society.^1^ These include changes in lifestyle habits, such as increased time spent at work, and consequently, greater exposure to irritating and antigenic substances. As a result, recent years have seen an increase in the frequency of occupational illnesses, defined as conditions acquired or developed as a result of work-related activities.^2^ Occupational illnesses are highly prevalent around the world, with occupational dermatoses (OD) being some of the most common diseases in this category. This is attributable to the fact that the skin is the largest organ in the body and is the most exposed to materials, tools, machinery and other aspects of the work environment.^3^ Contact dermatitis accounts for 4 to 7% of dermatology consultations, with half the cases attributed to occupational causes.^4^ According to current estimates, 60% of occupational illnesses in developing countries fall in the category of OD, with 1% of all workers thought to be affected by these issues.^5,6^ OD affect workers’ quality of life and productivity,^7^ and as such, a confirmed diagnosis can benefit workers by granting their access to legal rights and compensation, while also benefiting employers by reducing the costs of absenteeism, work leave and occupational health audits, as well as the time and money spent on examinations, diagnosis, treatment and rehabilitation.^8^ In the United States, the annual cost of OD is estimated to exceed US$ 1 billion.^5^ In Brazil and other developing countries, this has not been sufficiently studied.^7,9^

In order to further their understanding of this issue, the Brazilian Ministry of Health has implemented a mandatory reporting protocol for OD.^8^ Starting in 2002, all cases of OD treated in hospitals or health care centers were required to be reported to the National Network of Occupational Health Care (RENAST) through compulsory notification forms completed manually by trained professionals.^7^ Subsequently, the year 2006 saw the implementation of a new reporting system known as the Information System on Notifiable Diseases (SINAN). The SINAN is a nationwide platform linked to the Unified Health System (SUS); it is used in all cities across the country and encompasses all workers, in both the formal and informal sectors.^5^ In this system, OD are defined as alterations of the skin, mucous membranes and appendages that are directly or indirectly caused, maintained or aggravated by work-related factors.^8^ Any skin disease where occupational exposure was considered a primary cause or contributing factor is considered an OD.^10^

Although reporting is mandatory, these conditions are complex and difficult to assess, and as such, are often underdiagnosed.^11^ A large number of dermatoses are therefore absent from official statistics and are never referred to specialists, since many patients resort to self-treatment^3^; additionally, many workers choose not seek health care services out of fear of losing their jobs. As a result, even if cases are reported, the high rates of underdiagnosis interfere with data analysis.^5^ The impact of this illness also tends to be underestimated, since it is not fatal and mild cases are accepted as a “natural” consequence of work. However, OD can have major repercussions, ranging from functional limitations to the need to change jobs.^12^ The monitoring of medical records and the course of illness can help workers increase their well-being and awareness through epidemiological surveillance of the most common diseases.^11^

Therefore, the aim of this study was to perform an epidemiological survey of OD in the state of Espírito Santo, based on reports sent to the SINAN between January 2007 and December 2016, so as to describe the profile of affected workers, understand the relationship between work and dermatosis, identify major causes of this condition, and support more effective prevention strategies.

## METHOD

This was a descriptive, cross-sectional study of secondary public data available in the SINAN. Data were collected in 2019, covering the period from 2007 to 2016, since more recent information was not available in the SINAN at the time of the study. The information was then entered into Microsoft Excel 2016, using filters to select only records for the state of Espírito Santo. The analysis included comparisons between cities, reporting years, age groups, education levels, genders, causal factors, ethnicities, occupations, lesion sites and periods of absence for treatment. Data were described using frequencies and percentages. The incidence density (ID) was determined using the equation described by Miranda et al.^8^ According to the authors, the ID is calculated as the ratio of cases to the number of people exposed, multiplied by the duration of exposure. The denominator was calculated using data from the 2010 Brazilian Demographic Census^13^ which calculated population size approximately halfway through the sampling period. The incidence was presented as the number of cases per 106 workers per year. Therefore, the ID can be interpreted as the number of individuals affected per 106 workers.

$$\begin{array}{c} \mathrm{ID}=\mathrm{wor}\ker s\;\mathrm{with}\;\mathrm{OD}\times 106/\mathrm{size}\;\mathrm{of}\;\mathrm{workforce}\;\mathrm{per}\;\mathrm{the}\;2010\\ \mathrm{Census}\times 10\;\mathrm{years} \end{array}$$

## RESULTS

A total of 340 cases of OD were reported in the state of Espírito Santo from 2007 to 2016. This corresponds to 5.5% of the 6,131 cases reported in Brazil during this period. Table 1 shows the number of cases reported per city. Cases were reported in 20 of the 78 cities in the state of Espírito Santo, which corresponds to 25.6% of cities in the state. The city of Atílio Vivácqua reported 268 cases of OD, accounting for nearly 79% of all reports in the state. In the remaining cities, cases were infrequent and isolated, although an increase in numbers was seen in the capital, Vitória, starting in 2013. Table 1 also provides information on whether patients required medical leave. As can be seen in the table, this information was not reported in 17% of cases.

**Table 1:** Number of cases of occupational dermatosis reported to the Information System on Notifiable Diseases (SINAN) in cities in the state of Espírito Santo, from 2007 to 2016

City	2007	2008	2009	2010	2011	2012	2013	2014	2015	2016	Total	%
Afonso Cláudio	0	0	0	0	0	1	0	0	0	0	1	0.3
Água Doce do Norte	0	0	0	0	0	0	0	0	1	0	1	0.3
Alegre	1	0	0	0	0	0	0	0	0	0	1	0.3
Anchieta	0	0	0	0	0	0	0	0	0	2	2	0.6
Atílio Vivácqua	3	72	87	38	35	4	11	10	8	0	268	78.8
Cachoeiro de Itapemirim	0	0	0	0	1	0	0	0	0	0	1	0.3
Cariacica	0	0	0	0	0	0	0	0	2	1	3	0.9
Colatina	0	1	0	2	0	0	0	0	0	1	4	1.2
Guarapari	0	0	0	0	2	0	0	0	0	0	2	0.6
Iconha	0	0	0	0	5	0	0	0	0	0	5	1.5
Irupi	0	0	0	0	0	0	0	0	1	0	1	0.3
Jerônimo Monteiro	0	0	0	0	1	0	0	0	0	0	1	0.3
Mimoso do Sul	0	0	0	0	1	0	0	0	0	0	1	0.3
Montanha	0	0	0	0	0	0	1	0	0	0	1	0.3
Muniz Freire	0	0	0	0	0	0	0	2	0	0	2	0.6
Ponto Belo	0	0	0	0	0	0	0	0	0	1	1	0.3
Santa Maria de Jetibá	1	0	0	0	0	0	0	0	0	0	1	0.3
São Mateus	0	0	0	0	1	0	0	0	0	0	1	0.3
Vargem Alta	0	0	0	0	0	0	0	1	0	0	1	0.3
Vitória	0	0	2	0	0	0	4	5	19	12	42	12.4
Total	5	73	89	40	46	5	16	18	31	17	340	100.0
Medical leave	1	8	6	4	21	3	7	6	10	4	70	20.6
No medical leave	2	43	70	30	24	2	8	11	15	8	213	62.6
Not reported	2	22	13	6	1	0	1	1	6	5	57	16.8

Source: SINAN.

**Figure 1:**
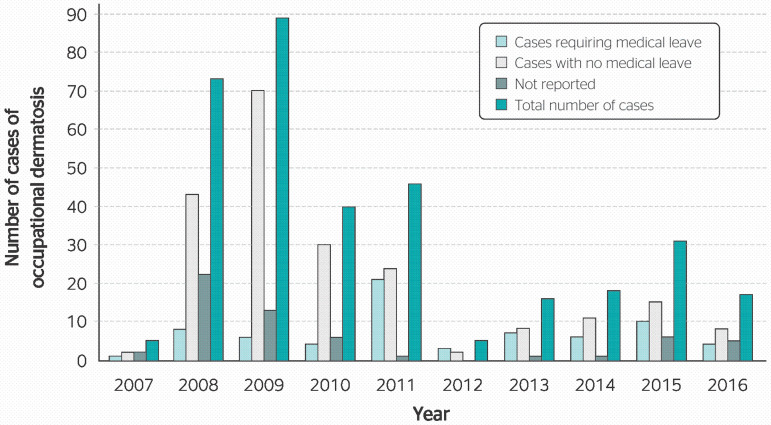
Annual number of cases of occupational dermatosis reported to the Information System on Notifiable Diseases (SINAN) in the state of Espírito Santo, from 2007 to 2016. Source: SINAN.

Figure 1 shows the number of cases reported per year, and illustrates a significant decrease in cases starting in 2012. The proportion of cases with and without medical leave is also shown in Figure 1. These results indicate that patients required medical leave in 25% of cases for which this information was available.

Table 2 shows the number of cases reported per age group and gender. The highest incidence was observed in individuals aged 20 to 29, and 30 to 39 years. Together, these two age groups accounted for over half the cases reported. However, percentage-wise, the highest ID was observed among individuals aged 70 years and older. Gender comparisons revealed a higher incidence in women: even though they comprise a smaller proportion of the workforce, the number of cases reported in women was higher than that reported in men.

**Table 2:** Gender and age of workers with occupational dermatosis in cases reported to the Information System on Notifiable Diseases (SINAN) in the state of Espírito Santo, from 2007 to 2016

Sex and age of affected workers	n	%	Worker population	ID
Gender				
Male	167	49.1	1,031,229	16.2
Female	173	50.9	796,204	21.7
Total	340	100.0	1,827,433	18.6
Age group (years)				
14 or younger	6	1.8	22,712	26.4
15-19	26	7.6	135,650	19.2
20-29	91	26.8	507,828	17.9
30-39	83	24.4	460,793	18.0
40-49	57	16.8	373,174	15.3
50-59	42	12.4	233,185	18.0
60-69	17	5.0	71,682	23.7
70 or more	12	3.5	22,409	53.5
Not reported	6	1.8	-	-
Total	340	100.0	1,827,433	18.6

Source: SINAN.

ID = Incidence density.

Table 3 shows the number of reports per education level and race/ethnicity. Although individuals with no formal education or incomplete primary school were more numerous in the workforce, the highest number of cases of OD was observed in individuals with complete primary and incomplete secondary education. These individuals accounted for 43.2% of total cases. The incidence in workers with higher education was significantly lower, at approximately 3%. The analysis of cases by race revealed that nearly 82% of workers affected by OD self-identified as being of Asian descent, while only 11% of cases involved workers who identified as white or brown.

**Table 3:** Education and race of workers with occupational dermatosis in cases reported to the Information System on Notifiable Diseases (SINAN) in the state of Espírito Santo, from 2007 to 2016

Gender and race of affected workers	n	%	Worker population	ID
Education				
No formal education and incomplete primary	52	15.3	707,429	7.4
Complete primary and incomplete secondary	147	43.2	332,572	44.2
Complete secondary and incomplete higher education	112	32.9	566,528	19.8
Complete higher education	11	3.2	213,025	5.2
Not reported	18	5.3	7,879	228.5
Total	340	100.0	1,827,433	18.6
Race				
White	35	10.3	785,941	4.5
Black	10	2.9	168,767	5.9
Asian	278	81.8	12,260	2267.5
Brown	2	0.6	855,266	0.2
Indigenous	1	0.3	5,199	19.2
Not reported	14	4.1	0	-
Total	340	100.0	1,827,433	18.6

Source: SINAN.

ID = Incidence density.

Table 4 shows the number of cases reported per occupation. Skilled workers, manual laborers, construction workers, mechanics and workers in similar occupations had the highest frequency of OD, with an ID of 44.8. This was followed by the unskilled workers, whose ID was 38.2. The lowest ID (6.5) was observed in service workers, shopkeepers and salespeople.

**Table 4:** Occupation of workers with occupational dermatosis in cases reported to the Information System on Notifiable Diseases (SINAN) in the state of Espírito Santo, from 2007 to 2016

Main occupation	n	%	Worker population	ID
Scientists and academics	16	4.7	143,729	11.1
Technical and mid-level workers	20	5.9	111,216	18.0
Administrative support workers	13	3.8	120,143	10.8
Service workers, shopkeepers and salespeople	18	5.3	276,279	6.5
Skilled workers in agriculture, forestry, hunting and fishing	20	5.9	156,966	12.7
Skilled workers, manual laborers, construction workers, mechanics and related occupations	88	25.9	196,479	44.8
Workers involved in machine assembly and installation	12	3.5	125,101	9.6
Unskilled workers	119	35.0	311,176	38.2
Poorly defined and undefined occupations	34	10.0	92,256	36.9
Total	340	100.0	1,538,087	22.1

Source: SINAN.

ID = Incidence density.

Table 5 shows the causes of illness and lesion sites for each case. Our results clearly showed that these items were rarely reported. Only five records (a little over 1% of the total) reported the site of the lesion. In two of these cases, the affected region was the eye. Low reporting rates were also observed with regard to the cause of illness, which was described in fewer than 15% of cases. Among cases for which this information was available, the most common etiologies were chemical agents, especially cleaning products such as chlorine, detergent, soap, disinfectants, and prolonged contact with rubber gloves, which were more common than physical or biological causes.

**Table 5:** Causes of occupational dermatosis in cases reported to the Information System on Notifiable Diseases (SINAN) in the state of Espírito Santo, from 2007 to 2016

Causal agents	n	%	Total	% in category
Agent category/description				
Chemicals			51	
Chlorine	5	1.5		9.8
Talcum powder	5	1.5		9.8
Latex, rubber and gloves	12	3.5		23.5
Detergents, disinfectants and soap	10	2.9		19.6
Unspecified cleaning products	2	0.6		3.9
Unspecified chemicals	3	0.9		5.9
Paper, fabric, leather and nylon	4	1.2		7.8
Ore, granite and tile	3	0.9		5.9
Chalk, plaster and quicklime	5	1.5		9.8
Metals, solvents and pesticides	2	0.6		3.9
Physical agents			4	
Blow	2	0.6		50.0
Motorcycle	1	0.3		25.0
Solder	1	0.3		25.0
Biological agents			5	
Plants	2	0.6		40.0
Microorganisms	2	0.6		40.0
Unspecified biological agent	1	0.3		20.0
Not reported	283	83.2	283	100.0
Lesion site				
Face	1	0.3		
Hands and neck	1	0.3		
Eyes	2	0.6		
Groin	1	0.3		
Not reported	335	98.5		

Source: SINAN.

## DISCUSSION

The comparison between the number of case reports (Table 1) and the population of each region in the country demonstrated that the state of Espírito Santo was overrepresented among cases of OD, accounting for 5.5% of reports but only 1.93% of the national population. However, the largest discrepancy in proportion of case reports and population size was observed in the city of Atílio Vivácqua. Contrary to the expectation that more populous cities would report the highest number of cases, the city of Atílio Vivácqua with only 11,335 inhabitants (0.3% of the state population) accounted for nearly 79% of case reports. The city’s economy is essentially agricultural, with an emphasis on dairy and coffee farms, although there has been an increase in the development of the ornamental stone sector due to the proximity of the mining center of Cachoeiro de Itapemirim.^14^ The occupation of affected workers and the causes of OD do not follow any particular pattern, and provide no explanation for these findings. The sharp decrease in the number of cases from 2012 onwards (Figure 1) is related to the worsening economic situation in Brazil. During this period, the national economy faced a major crisis and a massive increase in unemployment. The gross domestic product (GDP) fell from +1.9% in 2012 to -3.3% in 2016.^15^

Although child labor is illegal in Brazil, one case of OD was reported in a 6-year-old child (Table 2). In six additional cases, patient age was not informed or was described as a negative number, which is indicative of data entry errors. The present findings also revealed that the frequency of OD appeared to be higher at the extremes of the age range (i.e., in workers aged 70 and older or 14 and younger). This may indicate a correlation between inattention and the occurrence of OD.

The analysis of education levels (Table 3) revealed no correlations between this variable and the incidence of OD. In fact, it appears that the number of case reports was similarly low in both the highest and lowest education groups. Most of the cases involved workers with intermediate education levels, possibly because these individuals were more likely to have high-risk occupations. Another interesting point shown in Table 3 is the overrepresentation of workers with Asian descent, who accounted for nearly 82% of cases. Most of the Brazilian population identifies as white or brown, with fewer than 1% being of Asian descent. As such, it is likely that information on race was not carefully recorded in report forms.

As can be seen in Table 5, the occupational categories most affected by OD were skilled workers, manual laborers, construction workers, mechanics and workers in related occupations, as well as unskilled workers. The high number of cases in these populations may be due to the frequency of OD in bricklayers (72 cases) and domestic workers (95 cases). This data complements and supports the information on causal agents summarized in Table 5. Most incidents were caused by chemical agents, especially domestic cleaning products, which explains the high incidence of OD in domestic workers. Similarly, the high number of cases among bricklayers may be explained by exposure to cement, which has been identified in the literature as a major cause of OD.^16^ It should also be noted that, in our society, domestic workers and bricklayers are considered unskilled workers. The lack of occupational training also contributes to the occurrence of OD. The literature also shows that workers in the cleaning and construction sectors are among the most affected by OD.^8^

Although Table 5 shows that most reports did not indicate the cause of OD, chemical agents accounted for 85% of cases where this information was reported. This percentage is in line with the literature, which has noted that chemical agents are responsible for 80 to 90% of cases of OD.^8,9^ Rubber and detergents are among the most commonly reported causes of OD in the literature,^6^ and the data in Table 5 corroborate this observation. Information on the frequency of medical leave revealed that 25% of workers required a leave of absence as a result of their illness. This is a relatively high percentage, and is likely to have significant social and economic repercussions.

Workplace prevention strategies are the best methods to reduce the incidence and prevalence of OD. These include the use of personal protective equipment (PPE), risk identification, the installation of lavatories near workstations, worker orientation, rapid cleanup of areas contaminated by chemical agents, removal of irritating compounds and, if possible, substitution of potentially sensitizing substances.^17^ Once these strategies are implemented, they can be optimized to achieve maximum results using minimum resources based on the continuous analysis of SINAN reports. However, this is only possible if the information reported to the SINAN is as complete as possible. These data must also be periodically analyzed to develop additional strategies and manage control measures.

In conclusion, the data showed that the reports are missing information on important variables such as the cause and site of OD. Overall, the highest number of cases involved female workers aged 20 to 29 years, with complete primary and incomplete secondary education, in unskilled occupations such as domestic work, and were associated with exposure to chemical agents such as chlorine, detergents, disinfectants, talcum powder, and latex. To prevent further damage or compromise to workers’ health, and improve the monitoring and management of public data, it is important to invest in professional training on the correct and complete reporting of OD; the conduction of epidemiological studies; and the education of workers, especially those in the cleaning and construction sectors. There is also a need to address underreporting and intensify the demand for accurate reports given the social and financial repercussions of these issues on work-related processes.
